# High Therapeutic Efficacy of a New Survivin LSP-Cancer Vaccine Containing CD4^+^ and CD8^+^ T-Cell Epitopes

**DOI:** 10.3389/fonc.2018.00517

**Published:** 2018-11-13

**Authors:** Fanny Onodi, Chahrazed Maherzi-Mechalikh, Alice Mougel, Nadine Ben Hamouda, Charlotte Taboas, Fabien Gueugnon, Thi Tran, Herve Nozach, Elodie Marcon, Alain Gey, Magali Terme, Ahmed Bouzidi, Bernard Maillere, Jérôme Kerzerho, Eric Tartour, Corinne Tanchot

**Affiliations:** ^1^INSERM U970, PARCC (Paris-Cardiovascular Research Center), Paris, France; ^2^Faculté de Médecine, Université Paris Descartes, Sorbonne Paris Cité, Paris, France; ^3^Service d'immunologie Biologique, Hôpital Européen Georges Pompidou, AP-HP, Paris, France; ^4^VAXEAL Research, Evry, France; ^5^CEA-Saclay, Institut des Sciences du Vivant Frederic Joliot, Service d'Ingénierie Moléculaire des Protéines, Gif Sur Yvette, France

**Keywords:** cancer vaccine, immunotherapy, long synthetic peptide, T-cell responses, tumor associated survivin antigen

## Abstract

The efficacy of an antitumoral vaccine relies both on the choice of the antigen targeted and on its design. The tumor antigen survivin is an attractive target to develop therapeutic cancer vaccines because of its restricted over-expression and vital functions in most human tumors. Accordingly, several clinical trials targeting survivin in various cancer indications have been conducted. Most of them relied on short peptide-based vaccines and showed promising, but limited clinical results. In this study, we investigated the immunogenicity and therapeutic efficacy of a new long synthetic peptide (LSP)-based cancer vaccine targeting the tumor antigen survivin (SVX). This SVX vaccine is composed of three long synthetic peptides containing several CD4^+^ and CD8^+^ T-cell epitopes, which bind to various HLA class II and class I molecules. Studies in healthy individuals showed CD4^+^ and CD8^+^ T-cell immunogenicity of SVX peptides in human, irrespective of the individual's HLA types. Importantly, high frequencies of spontaneous T-cell precursors specific to SVX peptides were also detected in the blood of various cancer patients, demonstrating the absence of tolerance against these peptides. We then demonstrated SVX vaccine's high therapeutic efficacy against four different established murine tumor models, associated with its capacity to generate both specific cytotoxic CD8^+^ and multifunctional Th1 CD4^+^ T-cell responses. When tumors were eradicated, generated memory T-cell responses protected against rechallenge allowing long-term protection against relapses. Treatment with SVX vaccine was also found to reshape the tumor microenvironment by increasing the tumor infiltration of both CD4^+^ and CD8^+^ T cells but not Treg cells therefore tipping the balance toward a highly efficient immune response. These results highlight that this LSP-based SVX vaccine appears as a promising cancer vaccine and warrants its further clinical development.

## Introduction

Therapeutic vaccination aiming to stimulate tumor specific T-cell responses is a promising tool in antitumoral strategies. Hundreds of early phase clinical trials have already been performed so far but they showed only modest clinical effects (1–3). The main hurdles limiting efficacy of cancer vaccines have been identified: their weak immunogenicity, targeted tumor antigen often being self-antigens (4) or dispensable antigens, thus increasing the risk of immune tumor evasion by the loss of target antigen expression (5); their restricted indication to certain cancer types or patients, as they do not target broadly expressed tumor antigens or do not use promiscuous T-cell epitopes restricted to various HLA molecules; the difficulty in achieving the right balance of CD4^+^ and CD8^+^ T-cell responses required to induce effective anti-tumor immune responses and long-term protection against relapses since CD4^+^ T cells are required for CD8^+^ memory T-cell induction and maintenance (6–8). Finally, a major obstacle is the presence of immunosuppressive mechanisms in the tumor microenvironment (3, 9). Specifically, therapeutic vaccination can induce regulatory CD4^+^ T cells (Treg) limiting the vaccine efficacy (10–12).

To address these hurdles, personalized cancer vaccine based on mutant tumor antigens (neoantigens) (13), and vaccine targeting universal tumor antigens are very attractive approaches. As such, the inhibitor of apoptosis (IAP) protein survivin is an attractive target for anticancer immunotherapy, since it is a near universally over-expressed tumor antigen in human cancers, whereas its expression is low or undetectable in most differentiated adult tissues (14). Human transcriptome analysis reveals that survivin is the fourth most highly expressed transcript in human cancer cells when compared to normal cells (15). In multiple cancers, survivin expression is a marker of poor prognosis (16).

In addition, survivin exerts critical functions in tumor cells by contributing notably to apoptosis evasion, cell division, resistance to therapy (chemotherapy and radiotherapy) (17, 18) and angiogenesis (19). Therefore, a potential down-regulation or loss of survivin expression as a strategy of immune escape would severely impair tumor cell's survival capacity.

Accordingly, several clinical trials have been conducted with different vaccine candidates targeting survivin in multiple cancer indications. These studies showed promising but limited clinical results (20–22). We hypothesized that all these defects relied on the inability of these vaccines to induce both robust and balanced CD4^+^ and CD8^+^ T-cell responses as most of them are based on short peptides mainly focusing on CD8^+^ cytotoxic T lymphocytes (CTL) responses. Long synthetic peptides (LSPs) are a vaccine modality using 25- to 100-mer peptides that can contain both CD4^+^ and CD8^+^ T-cell epitopes. Several studies have demonstrated the relevance of LSP-based vaccines over short peptides or recombinant proteins to generate robust and long-term CD4^+^ and CD8^+^ T-cell responses, while limiting the induction of immune tolerance (7, 23–25). LSP-based vaccines were found to be safe, well-tolerated, and showed promising clinical efficacy in patients with pre-neoplastic lesions (3).

In this study, we thus designed a new survivin-based vaccine composed of three LSPs (SVX) encompassing multiple CD4^+^ and CD8^+^ T-cell epitopes. We evaluated the immunogenicity of the SVX peptides by conducting *in vitro* assays on human blood samples (from both healthy and cancer cell donors) and the therapeutic efficacy of the SVX vaccine *in vivo* in murine models using various established tumor cell lines.

## Materials and methods

### Peptides

SVX peptides (LSPs) derived from the native sequence of the human tumor antigen survivin (S1: 17–34; S2: 84–110; and S3: 122–142) (Table 1) were purchased from Almac Sciences.

**Table 1:** Position and amino acid sequence of the T-cell epitopes contained in the SVX vaccine.

**LSP name**	**CD4**^**+**^ **T-cell epitope**			**CD8**^**+**^ **T-cell epitope**		
	**Position**	**Sequence**	**HLA class II restriction**	**Position**	**Sequence**	**HLA class I restriction**
S1 (18aa)	17–31	HRISTFKNWPFLEGC	HLA-DR4, DR7, DR15, DRB4, DRB5, DP4	18-27	RISTFKNWPF	HLA-A3^a^
	20–34	STFKNWPFLEGCACT	HLA-DR4, DR7, DR11, DP4	18-28	RISTFKNWPFL	HLA-A2^a^
				20-28	STFKNWPFL	HLA-A24^b^
S2 (27aa)	84–98	CAFLSVKKQFEELTL	ND	92-101	QFEELTLGEF	HLA-A1^a^
	90–104	KKQFEELTLGEFLKL	HLA-DR7, DR11, DR15, DRB4, DRB5		
	93–107	FEELTLGEFLKLDRE	HLA-DR7, DP4	95-104	ELTLGEFLKL	HLA-A2^c, d^
	96–110	LTLGEFLKLDRERAK	HLA-DR4, DR7, DP4	96-104	LTLGEFLKL	HLA-A2^d^
S3 (21aa)	122–136	KEFEETAKKVRRAIE	—	—	—	—
	128–142	AKKVRRAIEQLAAMD	HLA-DR15, DRB5			

a−d *Correspond to references in paper (a) Reker et al. (26); (b) Andersen et al. (27); (c) Schmitz et al. (28); (d) Andersen et al. (29)*.

### Blood samples

Blood samples from 3 healthy donors and 35 cancer patients [10 lung cancers, 11 head & neck cancers, 14 metastatic renal cell carcinomas (mRCC)] were collected at the Hôpital Européen Georges Pompidou (Paris, France) after approval by the local ethics committee (CPP Ile de France, II N°2013-06-03). Blood samples were also collected from 12 healthy donors at the Etablissement Français du Sang (EFS, Rungis, France) after informed consent and following EFS guidelines. PBMCs were isolated by density gradient (Ficoll-Paque PLUS, GE Healthcare). The HLA-DR or A^*^02:01 genotypes of all donors were determined using the Gold SSP DRB1 typing kit (Invitrogen) after DNA extraction of PBMCs with NucleoSpin Blood L Kit (Macherey Nagel) or by staining with the monoclonal anti-HLA-A2-FITC antibody (Clone BB7.2, BD Biosciences).

### Generation and specificity of SVX-specific T-cell lines isolated from healthy donors

Monocyte-derived dendritic cells (DCs) generated as previously described (30) were loaded with the pool of survivin peptides (S1, S2, and S3, each at 5 μMol) and incubated for 4 h at 37°C.

For the induction of CD4^+^ T-cell lines, CD4^+^ T cells were isolated by positive selection using anti-CD4 mAbs coupled to magnetic microbeads (Miltenyi Biotec). Pulsed mDCs were washed and 1–3 × 10^4^ were added to each round-bottom microwell already containing 1–3 × 10^5^ autologous CD4^+^ T lymphocytes in 200 μL IMDM medium (Invitrogen) supplemented by 10% Human AB serum (Lonza), 1,000 U/mL rh-IL6 (R&D systems) and 10 ng/mL rh-IL12 (R&D Systems). The CD4^+^ T lymphocytes were restimulated on days 7, 14, and 21 with autologous mDCs freshly loaded with the pool of survivin peptides, and were grown in complete IMDM medium supplemented with 10 U/mL IL-2 (R&D systems) and 5 ng/mL IL-7 (R&D systems). The specificity of the CD4^+^ T-cell lines was assessed by IFN-γ enzyme-linked immunospot (ELISpot) on day 28 using autologous PBMCs loaded with the pool or individual SVX peptides.

For the induction of CD8^+^ T-cell lines, CD8^+^ T cells were isolated by positive selection using anti-CD8 mAbs coupled to magnetic microbeads (Miltenyi Biotec). Pulsed mDCs were washed and 2 × 10^4^ were added to each round-bottom microwell already containing 2 × 10^5^ autologous CD8^+^ T lymphocytes in 200 μL IMDM medium (Invitrogen) supplemented by 10% Human AB serum (Lonza), and 30 ng/mL IL-21 (R&D systems). After 3 days of culture, 5 ng/mL IL-7 and 5 ng/mL IL-15 (R&D systems) were added to the cultures. CD8^+^ T-cell lines were restimulated once on day 7 with 2 × 10^4^ autologous peptide-loaded DC and were grown in complete IMDM supplemented with IL-7 and IL-15 (5 ng/mL, each). The specificity of the CD8^+^ T-cell lines was assessed by IFN-γ ELISpot assay on day 14 using C1R-A2 cells loaded with the pool or the individual SVX peptides.

A response was considered positive if the number of spots per well obtained in peptide(s) stimulated conditions was two-fold higher than the number of spots counted without peptide(s), with a cut-off at 10 spot-forming cells after subtracting background.

### Assessment of survivin-specific T-cell responses in cancer patients

PBMCs were cultured for 7 days at 2 × 10^6^ cells/ml with the pool of SVX peptides (each peptide at a concentration of 10 μg/mL in complete RPMI 1640 medium supplemented with 5% serum AB). Cultured T cells were then added with SVX peptides to triplicate wells at 1 × 10^5^ cells/well in AIM V medium for 24 h at 37°C in 5% CO_2_. Plates were revealed using human IFN-γ ELISpot assays (Diaclone). A response was considered positive if the number of spots per well obtained in peptide(s) stimulated conditions was two-fold higher than the number of spots counted without peptide(s), with a cut-off at 10 spot-forming cells after subtracting background.

### Mice

Six- to ten-week-old BALB/c (H2^d^) (Charles River laboratory) and humanized HLA-A^*^0201/HLA-DR^*^0101 (HLA-A2/DR1) transgenic mice (obtained from Dr. Y.C. Lone) were used (31). All mice were kept under specific pathogen-free conditions at the INSERM U970 animal facility. All experiments have been approved by the local Paris-Descartes Ethics Committee for Animal Research (CEEA34.CT.143.12).

### Cell lines and tumor models

In BALB/c mice, different murine cell lines (from ATCC) have been used: a colorectal carcinoma (CT26) (MHC class I^+^II^−^), a B lymphoma (A20) (MHC class I^+^II^+^), a renal adenocarcinoma (Renca) (MHC class I^+^II^−^) cell lines. In the HLA-A2/DR1 mice, we used a sarcoma cell line (Sarc-A2) (kindly provided by Pr. O. Adotevi). The Sarc-A2 tumor cell line derives from a spontaneous arising sarcoma in HLA-A2/DR1 mice that was further transfected with the HLA-A2 molecules (32). To accurately evaluate the response directed against the human SVX peptides, all these tumor cell lines (expressing endogeneous murine survivin), were transfected after electroporation with a plasmid containing the whole human survivin sequence [pcDNA3-hsurvivin (pBir5^+^)] (30). Human-survivin expressing cells were then selected with 5 μg/mL of G418 (Invivogen). After several rounds of *in vitro* selection and amplification, the expression of human survivin was monitored by flow cytometry after intracellular staining with an anti-survivin PE antibody (BD Bioscience). hCT26 (2 × 10^5^ cells), hA20 (2.5 × 10^5^ cells), hRenca (5 × 10^5^ cells), and hSarc-A2 (5 × 10^5^ cells) were injected subcutaneously (s.c) into the right side of mice abdomen. Tumor growth was monitored twice a week using a caliper.

### Vaccine preparation and administration

Mice were vaccinated s.c in the abdomen with SVX vaccine (S1+S2+S3) (100 μg/peptide/mouse) adjuvanted with 50 μg of CpG (Litenimod, Oligovax SAS) emulsified in incomplete Freud's adjuvant (IFA, Sigma) and PBS 1X (Gibco) and boosted 2 weeks later with SVX (100 μg/peptide/mouse) without adjuvants. To test different adjuvant combinations, SVX was administered s.c with either 50 μg of CpG ± 20 μg of granulocyte macrophage colony stimulating factor (GM-CSF) (Peprotech), 50 μg of Poly ICLC (Oncovir), 100 nM KLK+ 4 nM ODN1a (IC31) (Intercell), 20 μg of Monophosphoryl lipid A (MPLA) (InvivoGen) or emulsified in IFA alone on BALB/c mice.

In tumor rejection assays, when tumor reached 10 mm2 (around day 5–day 7), mice were vaccinated s.c with SVX + CpG/IFA and boosted 1 week later with SVX.

For CD8^+^ T-cell depletion studies, 100 μg of anti-CD8 antibody (clone 2.43; BioXcell) or isotype control antibody (rat IgG2a) was administered i.p to tumor bearing mice the day before vaccination and each subsequent week. CD8 depletion was verified by flow cytometry.

### Assessment of survivin-specific T-cell responses in mice

Whole spleen cells were re-suspended at 2 × 10^6^ cells/mL in complete RPMI media. 2 × 10^5^ cells/well were then cultured in duplicate in 200 μL complete RPMI containing S1, S2, and/or S3 (each at 10 μg/mL), a well-described H2^d^-restricted CD8^+^ T-cell epitope surv85-93 (33) (10 μg/mL) or with tumor cell lines (after mitomycin C treatment, 50 μg/mL). Plates were incubated overnight at 37°C, 5% CO_2_ and developed the next day using murine IFN-γ ELISpot (Diaclone).

### IFN-*γ* elispot

Spots were counted using an ImmunoSpot analyzer (C.T.L) and enumerated as number of spot-forming cells per well. Cells incubated with medium alone or 100 ng/mL of phorbolmyristate acetate (PMA) and 500 ng/mL of ionomycin (Sigma Aldrich) were used as negative and positive controls, respectively. The number of specific T cells was calculated after subtracting negative control values. A response was considered positive if the number of spots per well-obtained in peptide(s) stimulated conditions was two-fold higher than the number of spots counted without peptide(s), with a cut-off at 10 spot-forming cells.

### Luminex

CD4^+^ T cells, isolated from spleen, were enriched by positive selection using magnetic beads (Miltenyi Biotec) and co-cultured with bone marrow derived DCs (BM-DC) as previously described (30). CD4^+^ T cells (2 × 10^5^ cells/well) were put in culture with 5 × 10^4^ DCs loaded or not (negative control) with S1+S2+S3 for 24 and 48 h at 37°C, 5% CO_2_. At 24 or 48 h after culture, supernatant was collected and ProcartaPlex Mouse Th1/Th2 Cytokine Panel (11 plex, eBioscience) was used to measure cytokines according to manufacturer's indications. The data was obtained using Bio-Plex® 200 (Biorad).

### Tumor dissociation

Tumors were extracted from mice upon termination and chopped into small pieces using a scalpel. Pieces were mechanically ground using a gentleMACS™ Dissociator (Miltenyi Biotec), filtered (40 μM) and treated with ACK solution. Cells were then washed in PBS and used for flow cytometry.

### Flow cytometry

Antibodies for surface staining against CD8 (53–6.7) (Ozyme), CD4 (RM4-5), CD3 (145-2C11), CD44 (IM7), CD62L (MEL-14), KLRG1 (2F1), PD-1 (J43) (eBioscience) were used.

For intracellular staining, samples were fixed/permeabilized (Foxp3/Transcription Factor Staining Buffer Set, eBioscience), and stained with FoxP3 (FJK-16s; eBioscience) and Granzyme B (GrB) (GB11; Ozyme) antibodies. Isotype controls were used as negative controls. Acquisitions were performed on a LSRII (BD Biosciences). The data were analyzed using FlowJo software (TreeStar).

### Statistical analysis

Results are expressed as means ± SEM. The Mann-Whitney test was used to compare two groups. The Kruskal–Wallis test was used to compare three or more groups. Comparison between tumor growth curves has been performed using a two-way ANOVA test and multiple comparisons have been corrected with the Bonferroni coefficient. Statistical significance was determined with Prism software (GraphPad Software). Significance was assumed at *P* < 0.05.

## Results

### Induction of CD4^+^ and CD8^+^ T-cell responses specific for SVX in healthy donors

**Figure 1:**
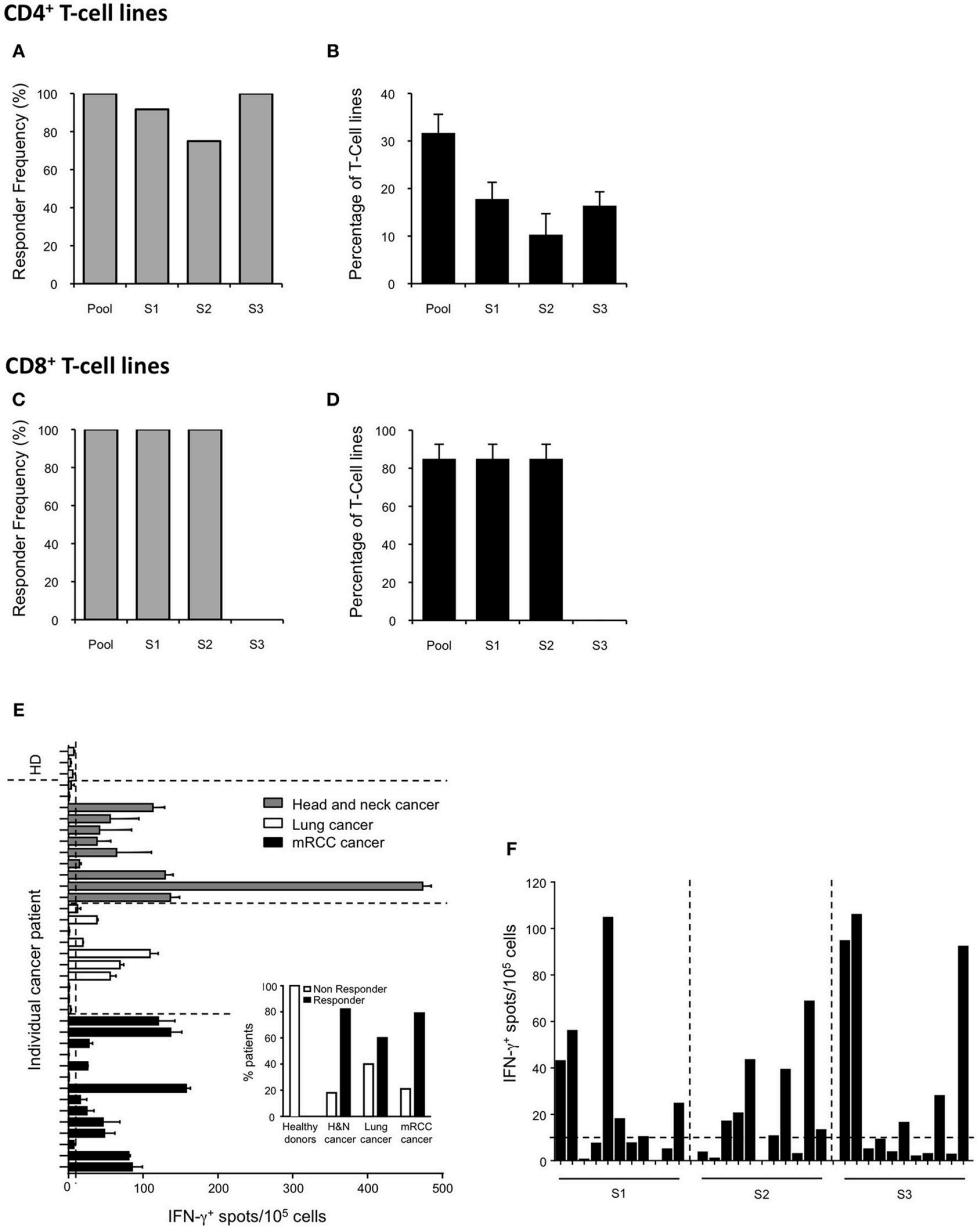
Induction of T-cell responses to SVX in healthy donors and spontaneous T-cell responses in cancer patients. **(A–D)** CD4^+^ and CD8^+^ T-cell responses against SVX peptides in healthy donors. CD4^+^ T cells from 12 healthy donors with diverse HLA-DRB1 genotype **(A,B)** or CD8^+^ T cells from 3 HLA-A*02:01 healthy donors **(C,D)** were repeatedly stimulated *in vitro* with the pool of SVX peptides (S1+S2+S3) loaded on autologous DCs. T-cell specificity was assessed by IFN-γ ELISpot assays using PBMCs or C1R-A2 loaded with the pool or individual SVX peptides. Histograms show the frequency of responding donors **(A,C)** and the mean percentages ± SEM of specific T-cell lines induced per donors **(B,D)** responding to each individual SVX peptide or at least one SVX peptide (Pool). **(E,F)** Spontaneous T-cell responses to SVX peptides in healthy donors, and cancer patients. **(E)** PBMC from 3 healthy donors, 11 head and neck, 10 lung, and 14 mRCC cancer patients were screened for spontaneous T-cell reactivity against the pool of SVX peptides, in IFN-γ ELISpot assays, after 1 week of *in vitro* culture with the pool of SVX peptides. **(E)** Intensity of the survivin response in different cancer patients and healthy donors. Each bar represents one patient or donor. Data are presented as means of IFN-γ spots from one experiment in triplicate. Small histograms represent the percentage of responder and of non-responder in healthy donors and in different types of cancer patients. **(F)** The 11 mRCC-responder patients were screened for spontaneous T-cell reactivity against individual SVX peptide, in IFN-γ ELISpot assays, after 1 week of *in vitro* culture. Data are presented as means of IFN-γ spots from one experiment in triplicate. A response was considered positive if the number of spots per well obtained in peptide(s) stimulated conditions was two-fold higher than the number of spots counted without peptide(s), with a cut-off at 10 spot-forming cells after subtracting background.

The SVX vaccine is composed of three long synthetic peptides (LSPs) derived from the native sequence of the human tumor antigen survivin (namely S1, S2, and S3) (Table 1). Collectively, these new LSPs contain 8 promiscuous CD4^+^ T-cell epitopes (30, 34) overlapping with 6 main described CD8^+^ T-cell epitopes restricted to HLA-A2, A3, A1, and A24 for which spontaneous CD8^+^ T-cell responses have been identified in numerous cancer patients (26–29, 35, 36) (Table 1). In order to anticipate the T-cell responses in vaccines as shown for a HIV LSP vaccine (37), the SVX peptides were introduced in long-term *in vitro* T-cell amplification assays using cells collected from healthy donors. We first quantified the CD4^+^ SVX-specific T-cell responses in 12 healthy donors with diverse HLA-DRB1 genotypes (Supplementary Table 1). Purified CD4^+^ T lymphocytes were seeded in multiple wells and stimulated weekly by autologous DCs previously loaded with the pool of SVX peptides. After three rounds of stimulation, the specificity of the T-cell lines (each well containing the expanded T cells) was tested by IFN-γ ELISpot assays. All donors generated a T-cell response specific for at least two of the three SVX peptides (Figure 1A and Supplementary Table 1). The SVX-specific CD4^+^ T-cell responses were highly directed against the S1 and S3 peptides, generating specific T-cell response in 91.7 and 100% of the donors respectively, while S2 peptide generated specific T-cell response in only 75% of the donors. We also determined the intensity of such responses by defining the percentage of positive T-cell lines for each patient (Figure 1B and Supplementary Table 1). An average of 31.7% ± 3.9 of T-cell lines contained specific CD4^+^ T lymphocytes against the pool of SVX peptides and 17.8% ± 3.5 and 16.4% ± 2.9 of T-cell lines contained S1 or S3 specific CD4^+^ T lymphocytes, respectively. S2 peptide was found to be slightly but not significantly less immunogenic than S1 and S3 as 10.3% ± 4.4 of T-cell lines contained S2-specific CD4^+^ T lymphocytes (Figure 1B).

The CD8^+^ T-cell response to SVX peptides was also evaluated in long-term *in vitro* T-cell amplification assays using CD8^+^ T cells harvested from HLA-A^*^ 02:01 positive healthy donors. All donors generated a specific T-cell response to the S1 and S2 peptides, which contain HLA-A2 restricted CD8^+^ T-cell epitopes (Table 1) (Figure 1C and Supplementary Table 2) but not to the S3 peptide which does not incorporate any HLA-A2 restricted CD8^+^ T-cell epitope. In addition, an average of 85% ± 7.6 of the seeded T-cell lines were found to contain S1 and S2 specific CD8^+^ T-cell lymphocytes, corresponding to a high level of immunogenicity (Figure 1D and Supplementary Table 2).

Collectively, these data demonstrated the high CD4^+^ and CD8^+^ T-cell immunogenicity of the SVX peptides in healthy individuals, regardless of the individual's HLA type and confirmed their potential in vaccination to generate survivin specific T-cell responses.

### Spontaneous T-cell responses against SVX peptides in selected cancer patients

In cancer patients, immune tolerance may preclude efficient T-cell activation. To evaluate the presence of pre-existing SVX-specific T-cell precursors in cancer patients, PBMCs (including both CD4^+^ and CD8^+^ T cells) of cancer patients (and healthy donors as controls) were cultured with the three SVX peptides for 1 week (short-term *in vitro* culture). Spontaneous T-cell responses to SVX peptides, assessed by IFN-γ ELISpot assays, were found in cancer patients [9/11 head and neck (82%), 6/10 lung (60%) and 11/14 (79%) renal cancers], but not in healthy donors (Figure 1E). These results demonstrate the presence of SVX specific T-cell precursors and the absence of immune tolerance against SVX peptides in most cancer patients studied. For the mRCC responder patients, specific spontaneous T-cell responses against each individual peptide have been monitored (Figure 1F). Interestingly the responses were heterogeneous between each patient, with some patients eliciting a specific T-cell response against one individual peptide (either S1, S2, or S3), some against two peptides and one patient against the three peptides. These results highlight that pooled together, the three peptides will elicit specific-SVX responses in a large fraction of cancer patients irrespective of their HLA's types. The demonstration that specific spontaneous responses against SVX peptides were detected in cancer patients was a prerequisite to develop a preclinical model to evaluate their potential as a vaccine.

### Survivin vaccine is immunogenic in mice

**Figure 2:**
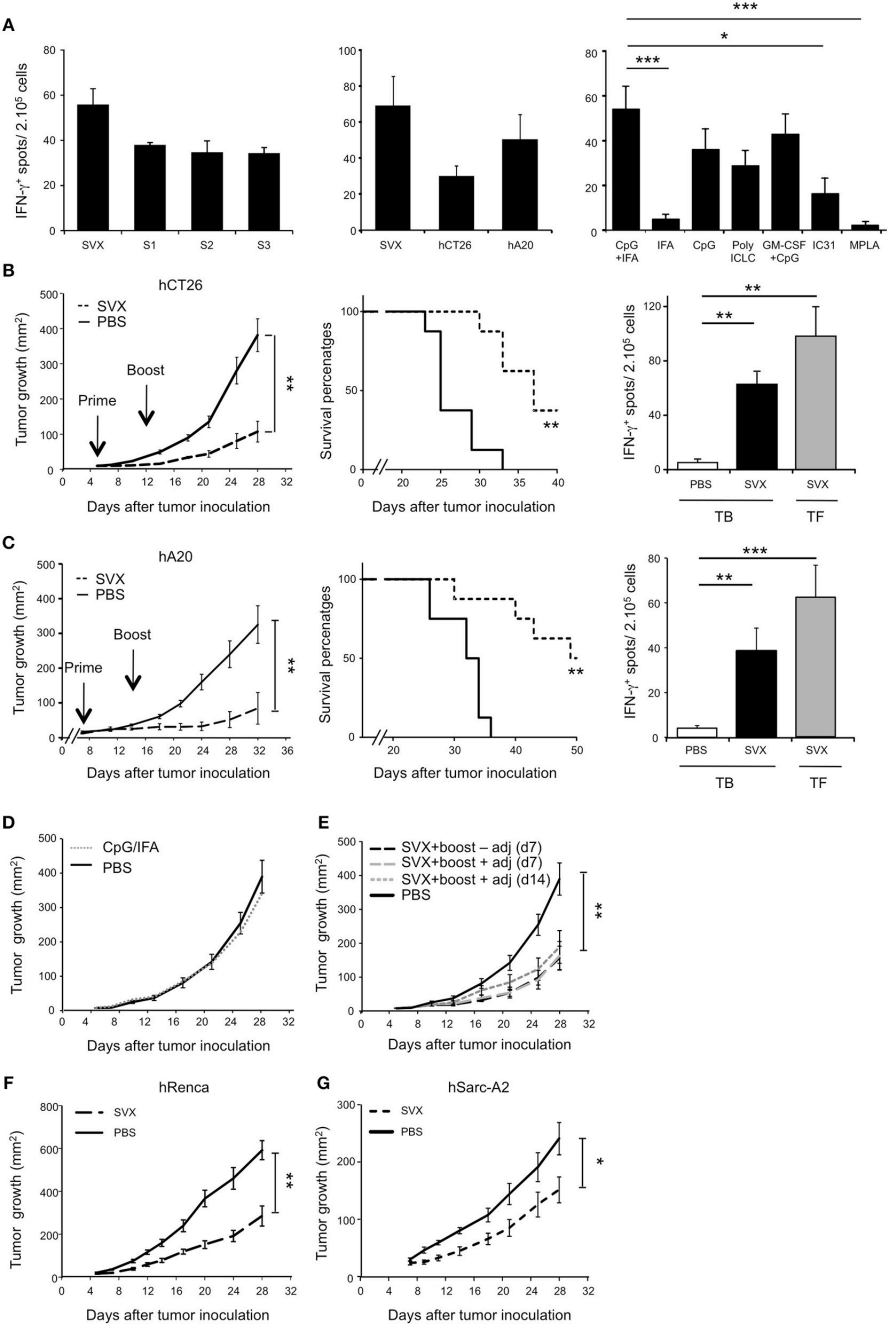
High therapeutic efficacy of SVX vaccine against various established tumor models**. (A)** T-cell immunogenicity of SVX vaccine and adjuvant selection. Tumor-free BALB/c (H2^d^) mice were subcutaneously (s.c) vaccinated as followed: priming with the three survivin LSPs (SVX) and adjuvants and boost 2 weeks later with SVX without adjuvant. One week after the boost, the induction of survivin-specific T-cell responses was analyzed by IFN-γ ELISpot assay on total splenocytes (2 × 10^5^ cells). Left and middle graphs showed overnight restimulation with the pool of SVX peptides or individual peptides (**A**, left graph) or tumor cell lines expressing the human survivin (hCT26 and hA20) (**A**, middle graph). Comparison of SVX specific T-cell responses induced by various adjuvants was performed by restimulation with the pool of SVX peptides (**A**, right graph). Results are the mean ± SEM of 5 mice per group and are representative of two to three independent experiments. ***P < 0.001, *P < 0.05. **(B,C)** Therapeutic experiments. BALB/c mice were engrafted s.c with hCT26 (2 × 10^5^ cells) **(B)** or hA20 cells (2.5 × 10^5^ cells) **(C)**. When tumors reached 10 mm^2^, mice were s.c injected with PBS, or immunized with SVX + CpG/IFA and received a boost 1 week later without adjuvant (SVX). **(B,C)** (Left graphs). Tumor growth was monitored twice a week and data are presented as mean tumor size (mm^2^) ± SEM. **P < 0.01. (Middle graphs). Kaplan-Meier survival curves of mice treated (dashed line) or not (black line) with SVX vaccine. The experimental endpoint was applied when tumor size reached 300 mm^2^. (Right Graphs). Intensity of SVX specific T-cell responses in the different groups: Tumor-bearing (TB) mice injected with PBS, or vaccinated (SVX) and Tumor-free (TF) mice immunized with SVX vaccine. Data are presented as means of IFN-γ spots ± SEM in the different groups of mice. **P < 0.01, ***P < 0.001. Data are representative of the results obtained in five separate experiments with 8 mice per group. **(D,E)** Control studies in the hCT26 tumor model. **(D)** Impact of the adjuvant on tumor growth. When tumors reached 10 mm^2^, mice were injected with PBS (PBS), or injected with the adjuvant alone (CpG/IFA). Data represent the mean of tumor size (mm^2^) ± SEM. **(E)** Impact of the vaccination strategy at boost on the vaccine efficacy. BALB/c mice were engrafted s.c with hCT26 tumor cells and injected with PBS (PBS), or vaccinated as followed: boost at d7 without adjuvant, at d7 with SVX + adjuvant or at d14 with SVX + adjuvant. Tumor growth was monitored every 2–3 days and data are presented as mean tumor size (mm^2^) ± SEM. The experiment has been repeated two-three times with similar results. **P < 0.01. **(F,G)** BALB/c mice engrafted s.c with hRenca (5 × 10^5^ cells) **(F)** or humanized HLA-A2/DR1 transgenic mice engrafted s.c with hSarc-A2 (5 × 10^5^ cells) tumor cells **(G)** were s.c injected with PBS or vaccinated with SVX + CpG/IFA and boosted 1 week later with SVX (SVX). Data are presented as mean tumor size (mm^2^) ± SEM and is representative of one out of two independent experiments with 8 mice per group. *P < 0.05, **P < 0.01.

Based on these results, we next performed preclinical vaccination in mouse models benefiting from the fact that the human and the mouse survivin proteins are highly conserved (84% identical and 91% homologous) (38). Importantly, the human SVX peptides contained highly conserved immunodominant epitopes described in mice, notably in H2^d^ background (33, 39, 40) (Supplementary Table 3) allowing evaluating the immune response induced by this new SVX vaccine in BALB/c mice. We first validated the immunogenicity of SVX vaccine following immunization in tumor-free BALB/c mice. Three weeks after vaccination, we performed an *in vitro* restimulation of splenocytes with either the mix of three LSPs or each individual peptide (Figure 2A, left graph). In all conditions, specific T-cell responses of similar intensity were observed as assessed by IFN-γ secretion, confirming the immunogenicity of SVX vaccine in mice. Similar results were obtained with murine tumor-cell lines expressing the human survivin (Figure 2A, middle graph) demonstrating that naturally processed epitopes were also recognized. Next, we studied the impact of different adjuvants or adjuvant combinations on the immunogenicity of SVX vaccine. T-cell immunogenicity of SVX vaccine was found to be significantly higher when formulated with CpG (TLR9), Poly ICLC (TLR3), or the combination of CpG with GM-CSF, compared to IFA, IC31, or MPLA (Figure 2A, right graph). The combination of CpG with IFA provided the highest immunogenicity of SVX vaccine and was thus selected as the optimal adjuvant combination.

### High therapeutic efficacy of SVX vaccine against various established tumor models

The therapeutic efficacy of the formulated SVX vaccine candidate was then evaluated in BALB/c mice engrafted with various syngeneic tumor models expressing the human survivin. Treatment with SVX vaccine was found to significantly inhibit the growth of established colorectal carcinoma (hCT26) (Figure 2B, left) and B-lymphoma (hA20) (Figure 2C, left) tumor cells compared to non-immunized mice or mice treated only with adjuvant combination (Figure 2D). To fully optimize vaccination's protocol, different strategies of boosting vaccination were investigated. Performing SVX boost + adjuvants (CpG/IFA) at day 7 or at day 14 provided similar efficacy as a boost without adjuvants at day 7 (Figure 2E). Inhibition of tumor growth was also observed with hRenca tumor cells in BALB/c mice or hSarc-A2 tumor model in HLA-A2/DR1 transgenic mice (Figures 2F,G). SVX vaccine also led to increased survival as 37.5% of mice were still alive at day 40 in the hCT26 model and 50% of mice still alive at day 50 in the hA20 model (Figures 2B,**C**, middle graphs).

This was associated with an induction of survivin-specific T-cell responses secreting high amounts of IFN-γ of similar intensity in vaccinated tumor-bearing (TB) and tumor-free (TF) mice (Figures 2B,C, right graphs).

**Figure 3:**
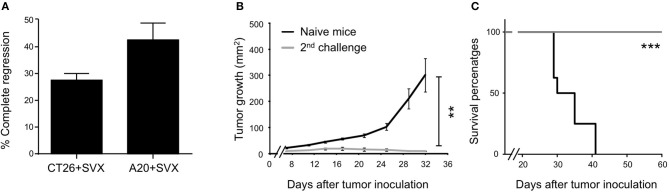
Generation of long term anti-tumor memory T cells following SVX vaccination. **(A–C)** Studies on tumor regression. **(A)** Histograms represent the percentage ± SEM of complete tumor regression of a pool of five different experiments (representing 40 mice) for both hCT26 and hA20 tumor models. **(B)** A group of vaccinated mice engrafted with hA20 cells (*n* = 5), which completely eliminated tumor cells were re-challenged with hA20 tumor cells. As positive control naive BALB/c mice (*n* = 5) were engrafted with hA20 tumor cells (Naive mice). Tumor growth **(B)** and Survival **(C)** were monitored. The experiment has been performed twice. **P < 0.01, ***P < 0.001.

Remarkably, over 5 experiments (representing 40 mice) performed in both hCT26 and hA20 models, treatment with SVX vaccine resulted in complete and sustained eradication of tumor in 27.5 and 42.5% of mice, respectively (Figure 3A). The capacity of SVX vaccine to induce anti-tumor memory immune responses was evaluated in all SVX-vaccinated mice that eradicated hA20 tumor cells in primary response. After an initial but small tumor growth, all animals completely eradicated tumor cells after a secondary challenge, compared to naive mice grafted with tumor cells (Figure 3B), leading to 100% of mice survival for more than 60 days (Figure 3C).

Altogether, these data demonstrate the high therapeutic efficacy of SVX vaccine against various established tumor cells, associated with its capacity to induce robust and specific T-cell responses but also effective memory T-cell responses for long-term protection against relapses.

### The therapeutic efficacy of SVX vaccine is mainly mediated by CD8^+^ T cells

**Figure 4:**
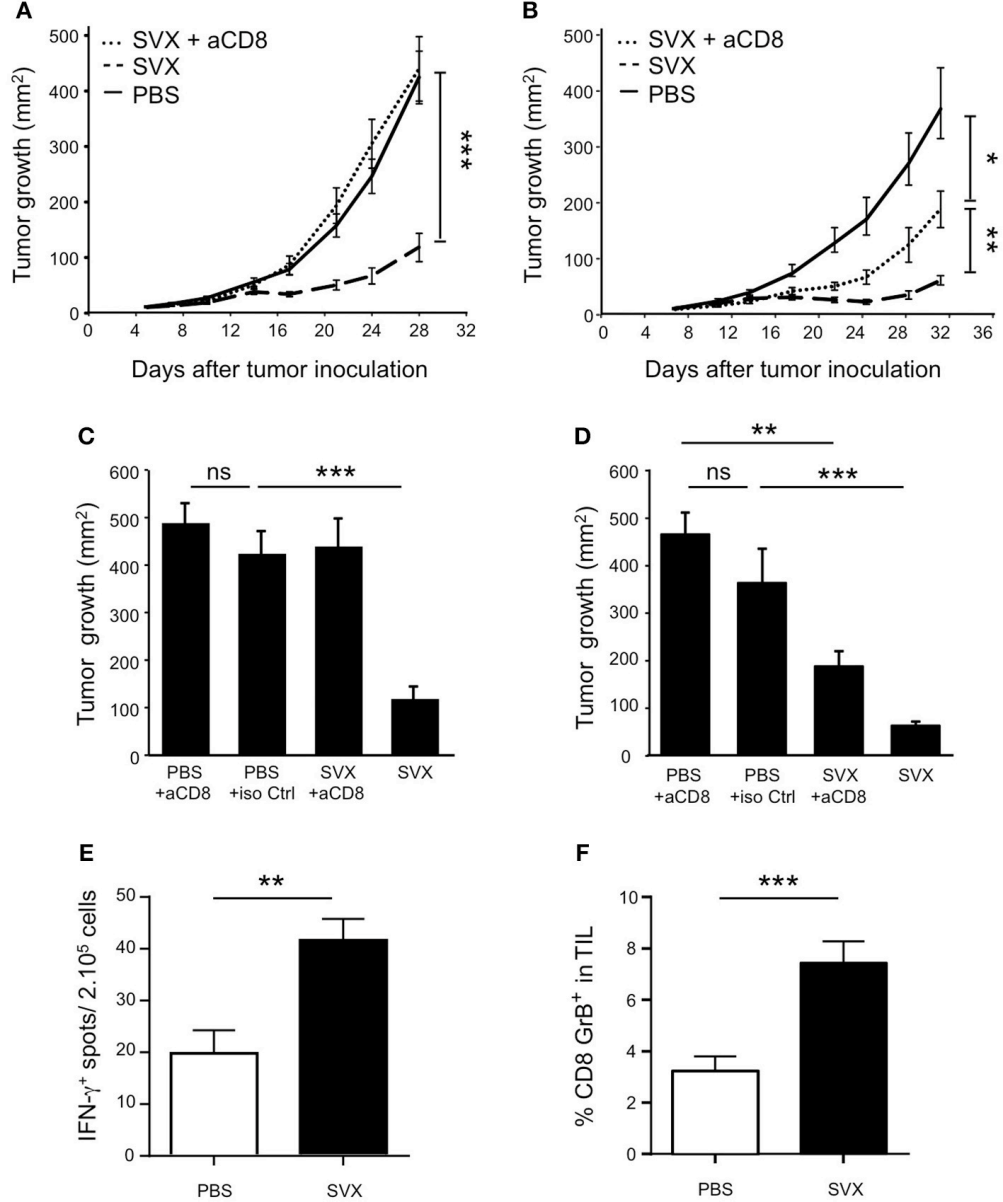
SVX therapeutic efficacy against tumor cells in CD8-depleted mice. BALB/c mice (8 mice per group) were engrafted s.c with hCT26 **(A,C)** or hA20 tumor cells **(B,D)**. When tumors reached 10 mm^2^, mice were s.c injected with PBS, or were immunized with SVX + CpG/IFA and received a boost 1 week later without adjuvant (SVX). **(A,B)** Groups of vaccinated mice engrafted with hCT26 **(A)** or hA20 tumor cells **(B)** were depleted of CD8^+^ T cells, using anti-CD8 mAbs (100 μg) injected intra-peritoneally (i.p) once a week, starting 1 day before SVX immunization (SVX + αCD8). Data are presented as mean tumor size (mm^2^) ± SEM from cohorts of 8 mice with *P < 0.05, **P < 0.01 and ***P < 0.001. Experiments have been done twice. **(C,D)** An additional group of mice for each experiment is shown and represents BALB/c mice (8 mice per group) engrafted with hCT26 **(C)** or hA20 tumors **(D)** and depleted of CD8^+^ T cells using anti-CD8 mAbs (100 μg) injected i.p once a week during 3 weeks. Data represent the mean of tumor size (mm^2^) ± SEM at day 28 **(C)** or day 32 **(D)**. **P < 0.01 and ***P < 0.001. **(E)** Intensity of survivin CD8^+^ specific T-cell responses in hCT26 TB mice vaccinated (SVX) or not with SVX (PBS). Evaluation of functional responses was performed 2 weeks after the last vaccination using IFN-γ ELISpot assays on total splenocytes restimulated overnight *in vitro* with the CD8^+^ T-cell epitope surv85-93. Data are presented as means of IFN-γ spots ± SEM of 16 mice per group from two independent experiments. **P < 0.01. **(F)** Same mice as in **(E)**. Two weeks after the last immunization, the tumors were harvested, and the percentage of CD8^+^GrB^+^ in TIL was evaluated by flow cytometry. Data are presented as means of percentage of cells ± SEM of 16 mice per group from two independent experiments. ***P < 0.001.

To evaluate the capacity of SVX vaccine to generate specific CD8^+^ T-cell responses and their role in tumor eradication, similar tumor rejection assays were performed in CD8-depleted mice. In absence of CD8^+^ T cells, the therapeutic efficacy of SVX vaccine was totally abolished against established hCT26 tumors (Figures 4A,C) while it was only partially impaired against hA20 tumors (Figures 4B,D). As a control, CD8 depleting treatment was also performed in non-vaccinated tumor-bearing (TB) animals. Similar tumor growths were observed in CD8-depleted and isotype control treated tumor-bearing animals, in both hCT26 (Figure 4C) and hA20 models (Figure 4D).

CD8^+^ T-cell responses against the surv85-93 epitope, a well-described CD8^+^ T-cell epitope in BALB/c mice (33, 39, 40), were also evaluated. Specific CD8^+^ T-cell responses, assessed by IFN-γ secretion, against this epitope were detected in SVX-vaccinated compared to non-vaccinated tumor-bearing mice (Figure 4E).

Finally, we monitored by flow cytometry, the expression of Granzyme B (GrB) by CD8^+^ T cells isolated from the tumor of SVX-vaccinated or not vaccinated mice. The percentage of GrB^+^ CD8^+^ T cells was found to be substantially higher among tumor infiltrating lymphocytes (TILs) isolated from vaccinated compared to non-vaccinated mice (Figure 4F).

Altogether, these results highlight the SVX vaccine's capacity to generate robust and effective anti-tumoral CTL responses and demonstrate their crucial role in its therapeutic efficacy against different MHC class I^+^ tumor cell types.

### SVX vaccine induces robust and specific Th1 responses

To evaluate the impact of CD4^+^ T-cell responses, we initially performed CD4 depletion in both hCT26 and hA20 models. CD4 depletion did not affect SVX vaccine efficacy in both vaccinated groups compared to non-depleted groups (Supplementary Figure 1). However, as CD4^+^ T cells are a heterogeneous population containing both T helper (Th) and Treg cells, the experimental protocol using total CD4 depletion is most likely inappropriate. To more accurately evaluate the role of CD4 T-cell help, we analyzed the cytokine profile of CD4^+^ T-cell responses induced with SVX vaccine.

**Figure 5:**
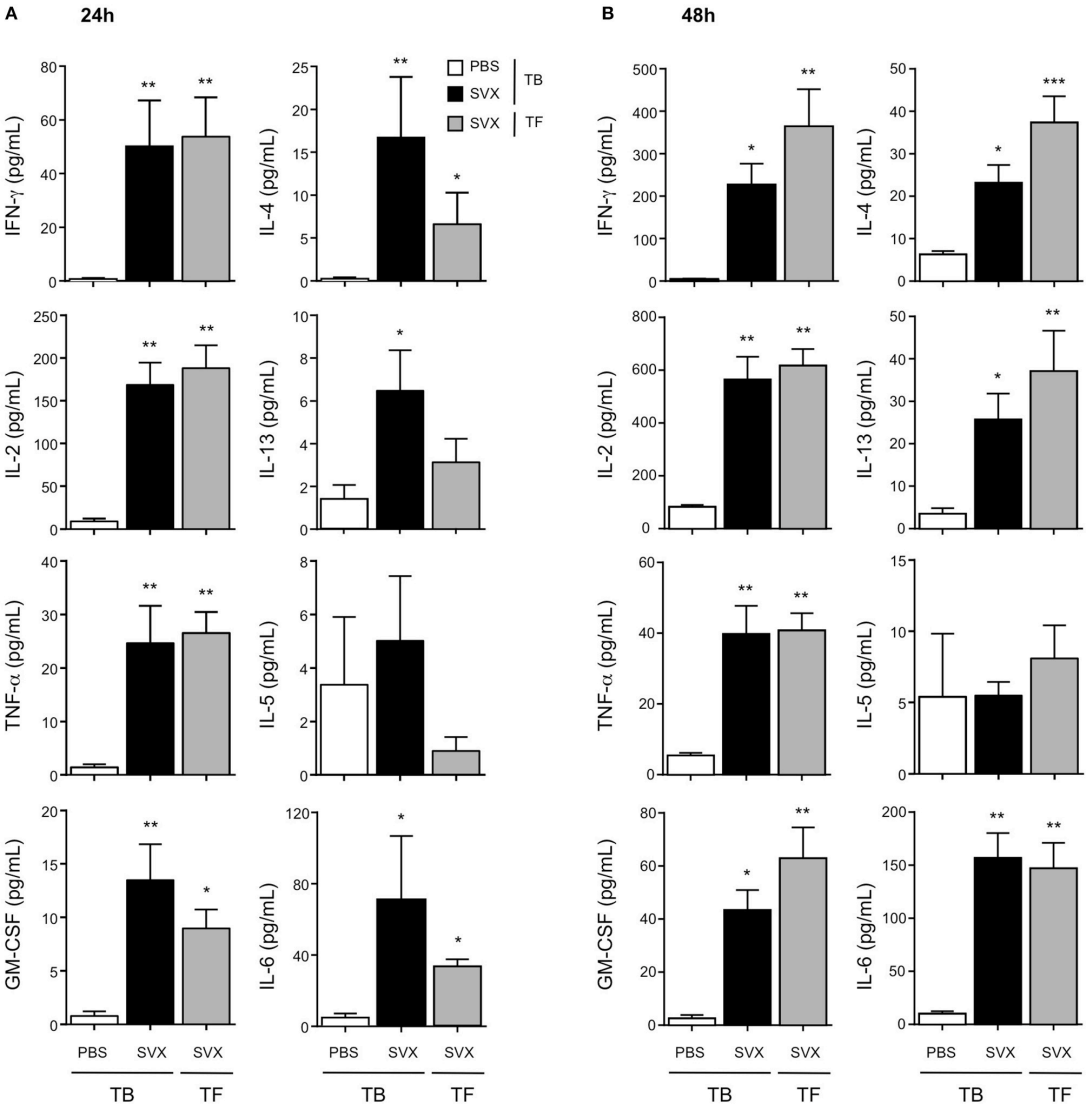
Cytokine profile of the CD4^+^ T-cell responses induced with SVX vaccine. Tumor-bearing (TB) mice engrafted s.c with hCT26 (*n* = 8 per group) were s.c injected with PBS or vaccinated with SVX vaccine (SVX). Tumor-free (TF) were immunized with SVX vaccine (SVX). Two weeks after the last immunization, splenic CD4^+^ T cells were cell sorted by magnetic beads. CD4^+^ T cells (2 × 10^5^) were then co-cultured with BM-DC (5 × 10^4^) pulsed with medium or the pool of SVX peptides. Cytokine productions were measured by Luminex assay performed on the supernatant after 24 h **(A)** or 48 h **(B)** of culture. Data are mean ± SEM of 8 mice per group with *P < 0.05, **P < 0.01, and ***P < 0.001.

After 24 h of *in vitro* restimulation, we predominantly detected CD4^+^ T cells with a Th1 profile, in both hCT26 tumor-bearing (TB) and tumor-free (TF) vaccinated groups, secreting IFN-γ, IL-2, TNF-α, and GM-CSF. CD4^+^ T cells isolated from non-vaccinated TB mice secreted limited levels of these cytokines (Figure 5A). The production of IFN-γ evaluated by ELISpot also showed similar level of IFN-γ secretion between vaccinated TB and TF mice (Supplementary Figure 2A). A same profile of cytokine secretion was observed in the B-lymphoma model (Supplementary Figures 2B,C). Cytokine production was also evaluated at 48 h in the hCT26 model. The level of cytokine secretion remained very low in non-vaccinated TB mice, while it was maintained and even increased in vaccinated groups. The production of IFN-γ, IL-2, and TNF-α was similarly increased in TB and TF vaccinated mice (Figure 5B).

Considering Th2 responses, low level of cytokine production was observed at 24 h (Figure 5A). At 48 h, CD4^+^ T cells isolated from vaccinated groups secreted significantly higher levels of IL-4 and IL-13 than those in non-vaccinated TB mice. However, low levels of IL-5 were detected in all groups (Figure 5B). SVX vaccine also induced some inflammatory cytokines such as IL-6 and IL-18 (data not shown), which could be detected in the supernatant of CD4^+^ T cells from vaccinated groups (Figures 5A,B).

Altogether these results demonstrate that SVX vaccine induces *in vivo* specific and multifunctional CD4^+^ T-cell responses with a predominant Th1 profile, not impacted by the presence of established tumors. This strong activation of CD4^+^ T cells could greatly participate to the high efficacy of the SVX vaccine by helping to induce and sustain CD8^+^ T-cell responses.

### SVX vaccine highly increase the ratio of both conventional CD4^+^ and CD8^+^ T cells over treg cells but has no significant impact on PD-1 expression

**Figure 6:**
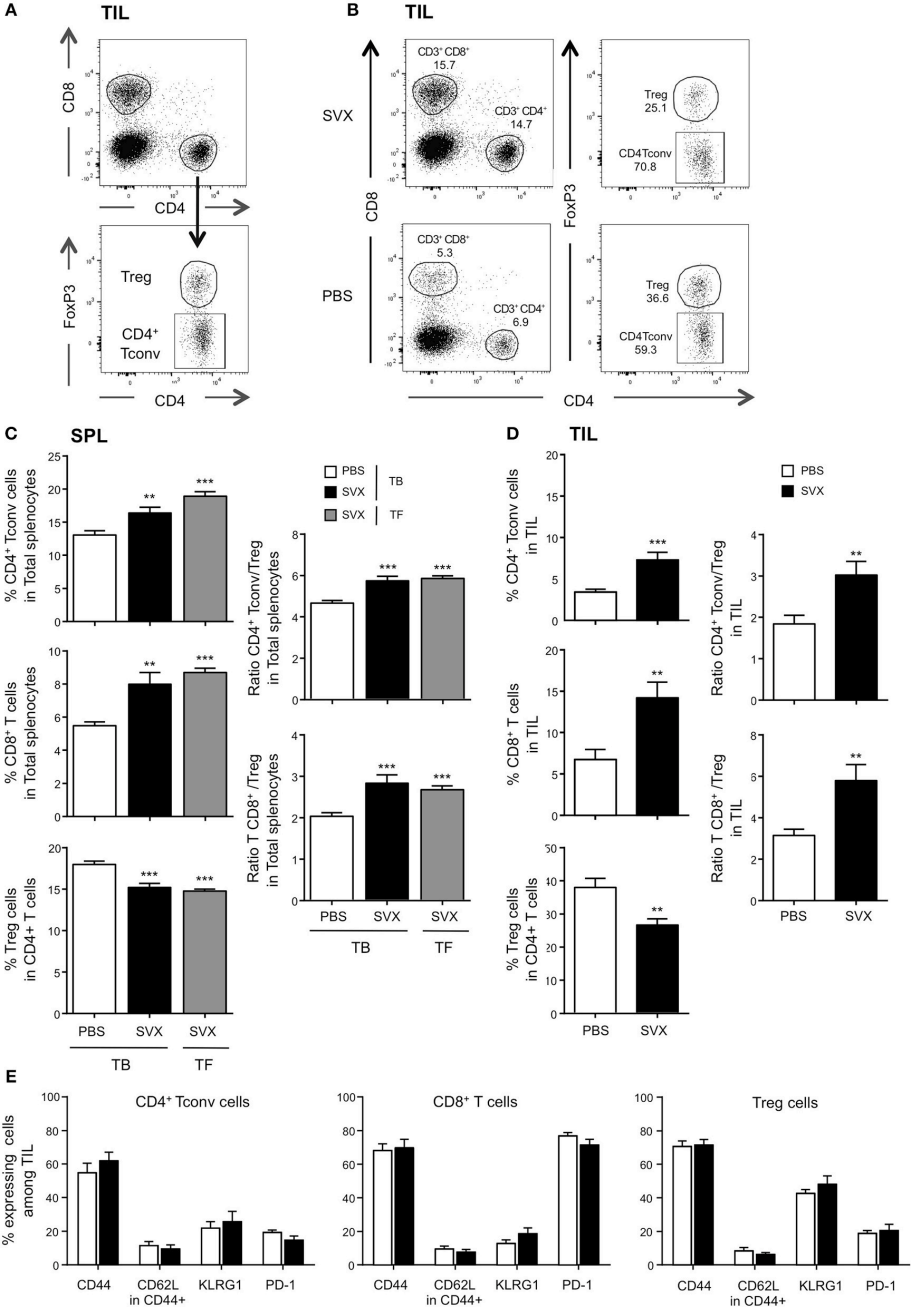
Impact of SVX vaccine on frequencies and phenotype of T-cell subsets both among splenocytes and TIL. **(A–E)** Groups of mice were the same as Figure 5. Two weeks after the last immunization, the spleen **(C)** and the tumor **(D)** were harvested, the immune cells were isolated, and the percentages of CD4^+^ Tconv, CD8^+^ T cells, and CD4^+^ FoxP3^+^ Treg cells were evaluated in each individual mouse by flow cytometry. **(A)** Dot plots show the strategy of gating to obtain the percentages of each population. **(B)** Dot plots show an example of percentage of each population recovered in TIL from TB mice vaccinated or not with SVX. The expression of different cell surface markers was also assessed in the different populations of T cells isolated from the tumor **(E)**. **(C,D)** (Left panels) Data are presented as means of percentage of cells ± SEM of 16 mice per group from two independent experiments. **(C,D)** (Right panels) The ratios of CD4^+^ Tconv/Treg and CD8/Treg of splenic **(C)** and TIL **(D)** were calculated for each individual mouse. Data are presented as means of ratios ± SEM of 16 mice per group from two independent experiments. **P < 0.01 and ***P < 0.001.

Among CD4^+^ T cells, the generation of immunosuppressive cells such as Treg cells may limit the efficacy of vaccine strategy. Using flow cytometry analysis, we thus evaluated the impact of SVX treatment on the frequencies and phenotypes of CD8^+^ T cells, CD4^+^ FoxP3^−^ Tconv, and CD4^+^ FoxP3^+^ Treg cells both in the spleen and among tumor infiltrating lymphocytes (TILs) (Figures 6A,B) of TB mice vaccinated or not with SVX but also in TF mice.

In SVX-vaccinated mice bearing hCT26 tumor, we observed significant increased frequencies of both splenic (Figure 6C) and intra-tumoral (Figure 6D) CD4^+^ Tconv and CD8^+^ T cells, and decreased frequencies of Treg cells. The effect was more pronounced within the tumor: two-fold increased in the vaccinated group compared to the non-vaccinated one. Similar results were observed in the spleen of TF vaccinated mice (Figure 6C). SVX vaccine thus favored high CD4^+^ Tconv/Treg and CD8/Treg ratios in both vaccinated groups compared to non-vaccinated TB animals (Figures 6C,**D**, right graphs).

Intra-tumoral T-cell populations generated upon SVX vaccination were also evaluated by assessing the expression of markers associated with central vs. effector memory differentiation (CD44, CD62L); KLRG1, a marker associated with better CD8^+^ anti-tumoral responses upon peptide vaccination in some studies (41) and PD-1, as a marker of T-cell activation and/or exhaustion. TILs expressed high level of CD44 but low level of CD62L (Figure 6E), identifying them as effector cells. KLRG1 was highly expressed on Treg, but mildly expressed on CD4^+^ Tconv and CD8^+^ T cells. Finally, low frequencies of PD-1 expressing CD4^+^ T cells and Treg, and high frequencies of PD-1 expressing CD8^+^ T cells (more than 70%) were detected among TILs of TB mice. Importantly, the phenotype of each cell subset was not different in vaccinated and non-vaccinated mice (Figure 6E). Similar observations were made in the hA20 model: a strong increase of intratumoral CD4^+^ Tconv/Treg and CD8/Treg ratios in vaccinated mice and no difference in PD-1 expression between vaccinated and non-vaccinated mice (Supplementary Figure 3).

Altogether, these results demonstrate the capacity of SVX vaccine to reshape the tumor microenvironment by strongly increasing the tumor infiltration of both CD4^+^ Tconv and CD8^+^ T cells over Treg cells therefore tipping the balance toward an efficient immune response.

## Discussion

A number of survivin-targeting immunotherapies have been developed so far including short peptide-based vaccines (20–22). These vaccines contained one or several HLA class I restricted survivin peptides and showed prolonged overall survival and induction of peptide specific CD8^+^ T-cell responses in various advanced cancers, with no adverse effects (42–46). However, CD8^+^ T-cell responses alone have not led to consistent clinical responses. Numerous studies have highlighted the fundamental role of CD4^+^ T cells in enhancing CD8^+^ T-cell responses and long term protection against relapses (20, 34, 47, 48). Combining CTL and CD4^+^ antigen-specific T-cell activation during vaccination may provide a theoretical advantage to improve survivin-targeted vaccination approaches. One study using such a vaccine strategy has been recently tested in a phase I trial in patients with malignant glioma (49). The survivin vaccine relies on the modified peptide SVN53-67/M57-KLH (SurVaxM) that contains antigen-binding motifs for multiple HLA class I and one potential HLA class II (50). This study showed that six of eight patients developed both cellular and humoral responses and had an overall survival of 86 vs. 25.7 weeks in an indirect comparative chemotherapy clinical trial (51), suggesting that such strategy may be highly beneficial.

In our study, we developed a more promiscuous survivin (SVX) vaccine, composed of three LSPs containing numerous CD4^+^ and CD8^+^ T-cell epitopes, presented to a large spectrum of HLA class II and I molecules. We first demonstrated the immunogenicity of the SVX peptides after long-term *in vitro* amplification of human PBMCs from healthy donors displaying either various HLA-DRB1 genotypes or HLA-A2 molecules for CD4^+^ and CD8^+^ T-cell responses, respectively: all tested donors responded to SVX peptides. These results unequivocally demonstrate that SVX vaccine includes immunogenic CD4^+^ and CD8^+^ T-cell epitopes. Because immune tolerance is a major hurdle in therapeutic vaccine strategies, we evaluated the efficacy of SVX peptides to stimulate T cells from the peripheral blood of cancer patients in short-term culture. We observed that 60–82% of cancer patients were responsive to SVX stimulation. These data suggested that SVX vaccine might boost specific T-cell responses in various cancer types and in a high proportion of patients. The presence of SVX T-cell precursors in cancer patients, that can be activated or re-activated, is crucial. Indeed, evaluating the efficacy of the vaccine only makes sense if targeted specific T cells are available. These results obtained in human were an important pre-requisite before going further and assessing the efficacy of the SVX vaccine in preclinical model. In BALB/c mice, we validated the therapeutic efficacy of SVX vaccine against different established tumor models. In all models tested, the reduction of tumor growth was associated with the induction of survivin specific T-cell responses. These results emphasize the broad potential of our therapeutic survivin-based vaccine approach.

The use of human survivin vaccine in murine models can be considered as a xeno-vaccination model that has been shown in some studies to generate higher immunogenicity that syngeneic vaccination (39, 52). Although the potential benefit of xeno-vaccination is debated (53, 54), it may be a limitation that needs to be taken into account in our model. However, we also performed studies in the humanized HLA-A2/DR1 transgenic mice, which more closely resemble human peptide presentation. In these mice, the SVX vaccine allowed a delay in the hSarc-A2 tumor growth, confirming the efficiency of the SVX vaccine in a human HLA context.

In BALB/c mice, the protection upon secondary challenge with tumor cells demonstrated that SVX vaccine was able to generate effective anti-tumor memory responses resulting in long-term protection against relapses. We next evaluated the impact of SVX vaccine on CD4^+^ and CD8^+^ T-cell responses. As expected, tumor rejection assays performed in CD8-depleted mice highlighted the capacity of SVX vaccine to induce strong anti-tumoral CD8^+^ T-cell responses. Interestingly, the impact of SVX vaccine on tumor growth was less strictly mediated by CD8^+^ T cells in the hA20 than in hCT26 tumor model, suggesting that other immune cells responsive to the survivin vaccine may participate to tumor control. In contrast, CD4^+^ T-cell depletion failed to demonstrate any impact on tumor growth, presumably due to the opposite effects provided by Th and Treg CD4^+^ T cells, respectively. When addressing directly CD4^+^ T-cell functions, CD4^+^ T cells exhibited multifunctional cytokine responses to SVX peptides, with a predominant Th1 profile in vaccinated tumor-bearing animals. These strong CD4^+^ T-cell responses may greatly participate to the efficacy of the survivin vaccine, in particular, in helping effective CD8^+^ memory T-cell generation. Because CD4^+^ T-cell responses are heterogeneous, we also examined Treg proportions following SVX vaccination in spleen and tumors. We demonstrated that SVX vaccine highly increased tumor infiltration by both CD4^+^ Tconv and CD8^+^ T cells. Importantly there is no increase in the percentage of Treg cells as previously described in some therapeutic vaccine designs (10–12). Consequently, the SVX vaccine appears to favor the balance toward strong effector immune responses. In addition to the analysis of T-cell compartment, the evaluation of the myeloid-derived suppressor cells (MDSCs) and tumor associated macrophages (TAMs) populations in the tumor microenvironment would also be an important issue to address, as they play a major role in tumor-related immunosuppression and can hamper successful immunotherapy approaches (55, 56).

Whereas, therapeutic vaccines may increase PD-1 expression on TILs (57, 58), we did not observed such increase in our study. Similar percentage of PD-1 expressing CD8^+^ TILs was observed between SVX-vaccinated and non-vaccinated animals. SVX vaccine might thus not be able to overcome all the immunosuppressive mechanisms developing during tumor development. These observations suggest that the combination of SVX vaccine with immune checkpoint blockade antibodies targeting PD-1 may potentially further increases its therapeutic efficacy against such established tumor cells, a hypothesis that we are currently addressing.

In summary, the SVX vaccine allowed to generate both CD4^+^ and CD8^+^ specific T-cell responses, leading in mice to high therapeutic efficacy in various tumor models. The SVX vaccine was also able to generate anti-tumor memory T-cell responses. Additionally, limited Treg expansion was observed, thus minoring Treg immunosuppressive effects. In humans, our assays on blood samples demonstrated that the SVX peptides were able to elicit specific T-cell responses in various cancer types and in a high proportion of patients. This highlights the absence of immune tolerance against the SVX peptides. Based on this set of experiments, SVX vaccine may constitute a very promising new vaccine candidate for clinical trials that could be used in a large spectrum of patients suffering from different cancers, and irrespectively of their HLA types.

## Author contributions

Conception and design: BM, JK, ET, and CoT. Development of methodology, generation, and specificity of SVX-specific T cell lines: FG, HN, EM, and BM. Development of methodology and design of the SVX vaccine: JK, AB, and BM. Acquisition of data (provided animals, acquired and managed patients, provided facilities, etc.): FO, CM-M, AM, ChT, and TT (animal studies); NB and AG (human studies), FG, HN, and EM (human cell lines). Analysis and interpretation of data (e.g., statistical analysis, biostatistics, computational analysis): FO, CM-M, AM, MT, JK, BM, and CoT. Writing, review, and/or revision of the manuscript: MT, JK, BM, ET, and CoT. Study supervision: BM and CoT.

### Conflict of interest statement

The authors declare that the research was conducted in the absence of any commercial or financial relationships that could be construed as a potential conflict of interest.
