# Supplementary material for: High Therapeutic Efficacy of a New Survivin LSP-Cancer Vaccine Containing CD4+ and CD8+ T-Cell Epitopes
Source: Front Oncol. 2018 Nov 13;8:517. doi: 10.3389/fonc.2018.00517 (PMC6243131; doi:10.3389/fonc.2018.00517)
Supplement: Supplementary file 1 [file Data_Sheet_1.PDF]

Supplementary material

High therapeutic efficacy of a new survivin LSP-cancer vaccine containing CD4$^+$ and CD8$^+$ T-cell epitopes

F. Onodi[1#], Maherzi C[1,2#], Mougel A[1,2], Ben Hamouda N[1,3], Taboas C[1], Gueugnon F[4,5], Tran T[1], Nozach H[5], Marcon E[5], Gey A[1,3], Terme M[1,2], Bouzidi A[4], Maillere B[5], Kerzerho J[4], Tartour E[1-3] and C. Tanchot[1*]

[#]both authors contributed equally to the paper.

[1] INSERM U970, PARCC (Paris-Cardiovascular Research Center); Paris, France.

[2] Faculté de Médecine, Université Paris Descartes, Sorbonne Paris Cité; Paris, France.

[3] Service d'immunologie biologique, Hôpital Européen Georges Pompidou, AP-HP; Paris, France.

[4] VAXEAL Research, Evry, France.

[5] CEA-Saclay, Institut des Sciences du Vivant Frederic Joliot, Service d'Ingénierie Moléculaire des Protéines ; Gif Sur Yvette F-91191, France.

[*]**Correspondence:**
Dr. Corinne Tanchot
corinne.tanchot@inserm.fr

1 Supplementary Tables

		Specific responses of $CD4^+$ T-cell lines to SVX Peptides			
**Donor**	**HLA-DRB1 Genotype**	**S1**	**S2**	**S3**	**Pool**
D1	07 :01/11 :01	13.3	6.7	6.7	23.3
D2	01 :01/03 :01	13.3	0.0	26.7	40.0
D3	04 :01/07 :01	10.0	3.3	16.7	23.3
D4	13 :01/14 :01	10.0	3.3	10.0	20.0
D5	04 :01/07 :01	0.0	13.3	10.0	20.0
D6	01 :01/12 :01	10.0	0.0	10.0	16.7
D7	01 :01/15 :01	40.0	53.3	40.0	60.0
D8	04 :01/15 :01	23.3	0.0	16.7	26.7
D9	07 :01/16 :01	30.0	3.3	13.3	30.0
D10	11 :01/15 :01	36.7	6.7	23.3	50.0
D11	08 :01/13 :01	13.3	23.3	20.0	43.3
D12	03 :01/04 :01	13.3	10.0	3.3	26.7
**Mean Response Intensity (%) ***		17.8+/-3.5	10.3+/-4.4	16.4+/-2.9	31.7+/-3.9
**Responder Frequency (%) #**		91.7	75	100	100

Supplementary Table 1. Specificity and magnitude of $CD4^+$ T-cell response against SVX peptides in healthy donors. Table shows numerical data used for the graphs in Figure 1A and 1B. It represents the analysis of $CD4^+$ T-cell lines from 12 healthy donors, with diverse HLA-DRB1 genotype. Thirty $CD4^+$ T-cell lines were seeded for each priming condition. $CD4^+$ T-cell specificity for SVX peptides was assessed by IFN-g ELISpot assays using PBMCs loaded with the pool or individual SVX peptides. A response was considered positive if the number of spots per well obtained in peptide(s) stimulated conditions was 2-fold higher than the number of spots counted without peptide(s), with a cut-off at 10 spot-forming cells after subtracting background. * Mean percentages ± SEM of specific $CD4^+$ T-cell lines induced per donors responding to the selected SVX peptide(s) in long-term T-cell amplification assays; # Percentage of healthy donors responding to the pool or one SVX peptide among 12 tested donors in long-term T-cell amplification assays. D1 to D12 = donor 1 to donor 12.

Donor	Specific responses of CD8+ T-cell lines to SVX Peptides			
	S1	S2	S3	Total
d1	100	100	0	100
d2	75	75	0	75
d3	80	80	0	80
**Mean Resp. Intensity (%) ***	85+/-7.6	85+/-7.6	0	85+/-7.6
**Resp. Frequency (%) #**	100	100	0	100

Supplementary Table 2. Specificity and magnitude of $CD8^+$ T-cell response against SVX peptides in healthy donors. Table shows numerical data used for the graphs in Figure 1C and 1D. It represents the analysis of $CD8^+$ T-cell lines from 3 HLA-A*02:01 healthy donors. Twenty $CD8^+$ T-cell lines were seeded for each priming condition. $CD8^+$ T-cell specificity for SVX peptides was assessed by IFN-γ ELISpot assays using C1R-A2 loaded with the pool or individual peptides. A response was considered positive if the number of spots per well obtained in peptide(s) stimulated conditions was 2-fold higher than the number of spots counted without peptide(s), with a cut-off at 10 spot-forming cells after subtracting background. * Mean percentages ± SEM of specific $CD8^+$ T-cell lines induced per donors responding to the selected SVX peptide(s) in long-term T-cell amplification assays; # Percentage of healthy donors responding to the pool or one SVX peptide among 3 tested donors in long-term T-cell amplification assays. d1 to d3 = donor 1 to donor 3.

1- Human Survivin peptides

Name	aa position	aa sequence	aa number
hS1	17-34	HRISTFKNWPFLEGCACT	18 AA
hS2	84-110	CAFLSVKKQFEELTLGEFLKLDRERAK	27 AA
hS3	128-142	AKKVRRAIEQLAAMD	15 AA

2- Homology to corresponding Mouse Survivin peptides

Name	aa position	aa sequence	aa number	Percentage of homology
mS1	17-34	**Y**RI**A**TFKNWPFLE**D**CACT	18 AA	84%
mS2	84-110	CAFL**T**VKKQ**M**EELT**VS**EFLKLDR**Q**RAK	27 AA	89%
mS3	128-142	AK**TTRQS**IEQLAA--	13 AA	80%

Supplementary Table 3. Amino acid sequences of human and mouse survivin protein. The human and mouse amino acid sequence share 84% of homology. Upper Table shows the amino acid sequences of human peptides composing the survivin vaccine. Lower table shows the amino acid sequence of corresponding mouse survivin peptides and their percentage of homology with human peptides. In Balb/C haplotype, S1 and S2 peptides contain a well-described Surv17-28 $CD4^+$ T-cell epitope and Surv85-93 $CD8^+$ T-cell epitope, respectively.

2 Supplementary Figures

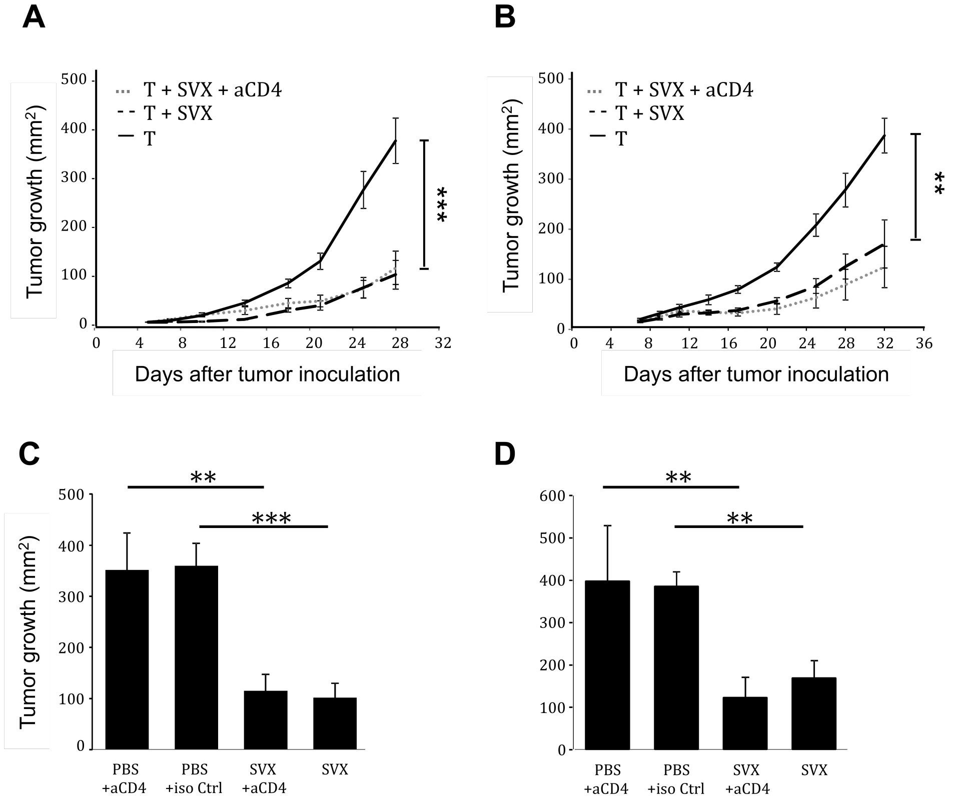

Supplementary Figure 1. CD4$^+$ T-cell depletion does not alter SVX vaccine efficacy. BALB/c mice (8 mice per group) were engrafted s.c with hCT26 (**A,C**) or hA20 tumor cells (**B,D**). When tumors reached 10 mm^2, mice were s.c injected with PBS, or were immunized with SVX + CpG/IFA and received a boost one week later without adjuvant (SVX). Groups of vaccinated mice engrafted with hCT26 (**A**) or hA20 tumor cells (**B**) were depleted of CD4$^+$ T cells, using anti-CD4 mAbs (200µg) injected intra-peritoneally (i.p) once a week, starting one day before SVX immunization(SVX + αCD4). Data are presented as mean tumor size (mm^2) ± SEM from cohorts of 8 mice with **P<0.01 and *** P<0.001. Experiments have been performed twice. (**C,D**). An additional group of mice for each experiment is shown and represents BALB/c mice (8 mice per group) engrafted with hCT26 (**C**) or hA20 tumors (**D**) and depleted of CD4$^+$ T cells, using anti-CD4 mAbs (200µg) injected i.p once a week during three weeks. Histograms represent the mean of tumor size (mm^2) ± SEM at day 28 (**C**) or day 32 (**D**). **P<0.01 and *** P<0.001. Experiments have been done twice.

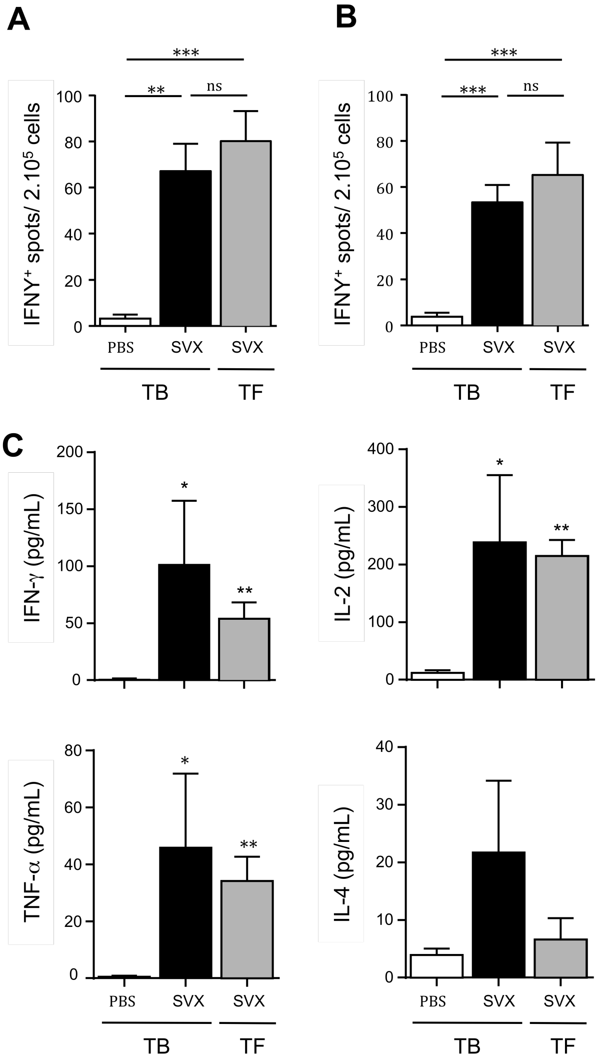

Supplementary Figure 2. SVX vaccine induces multi-functional $CD4^+$ T-cell responses in both hCT26 and hA20 tumor-bearing mice. Tumor-bearing (TB) mice engrafted s.c with hCT26 (n=8 per group) **(A)** or hA20 tumor cells (n=8) **(B,C)** and s.c injected with PBS or vaccinated with SVX (SVX)**.** Tumor-free (TF) mice were immunized with SVX vaccine (SVX). Two weeks after the last immunization, splenic $CD4^+$ T cells were cell sorted by magnetic beads. $CD4^+$ T cells ($2x10^5$) were then co-cultured with BM-DC ($5x10^4$) pulsed with medium or the pool of SVX peptides. **(A,B)**. The intensity of SVX-specific $CD4^+$ T-cell responses was evaluated using IFN-γ ELISpot assays. **(C).** Cytokines were measured by Luminex assay performed on the supernatant after 24h of culture. Data are mean ± SEM of 8 mice per group with *P<0.05, **P<0.01.

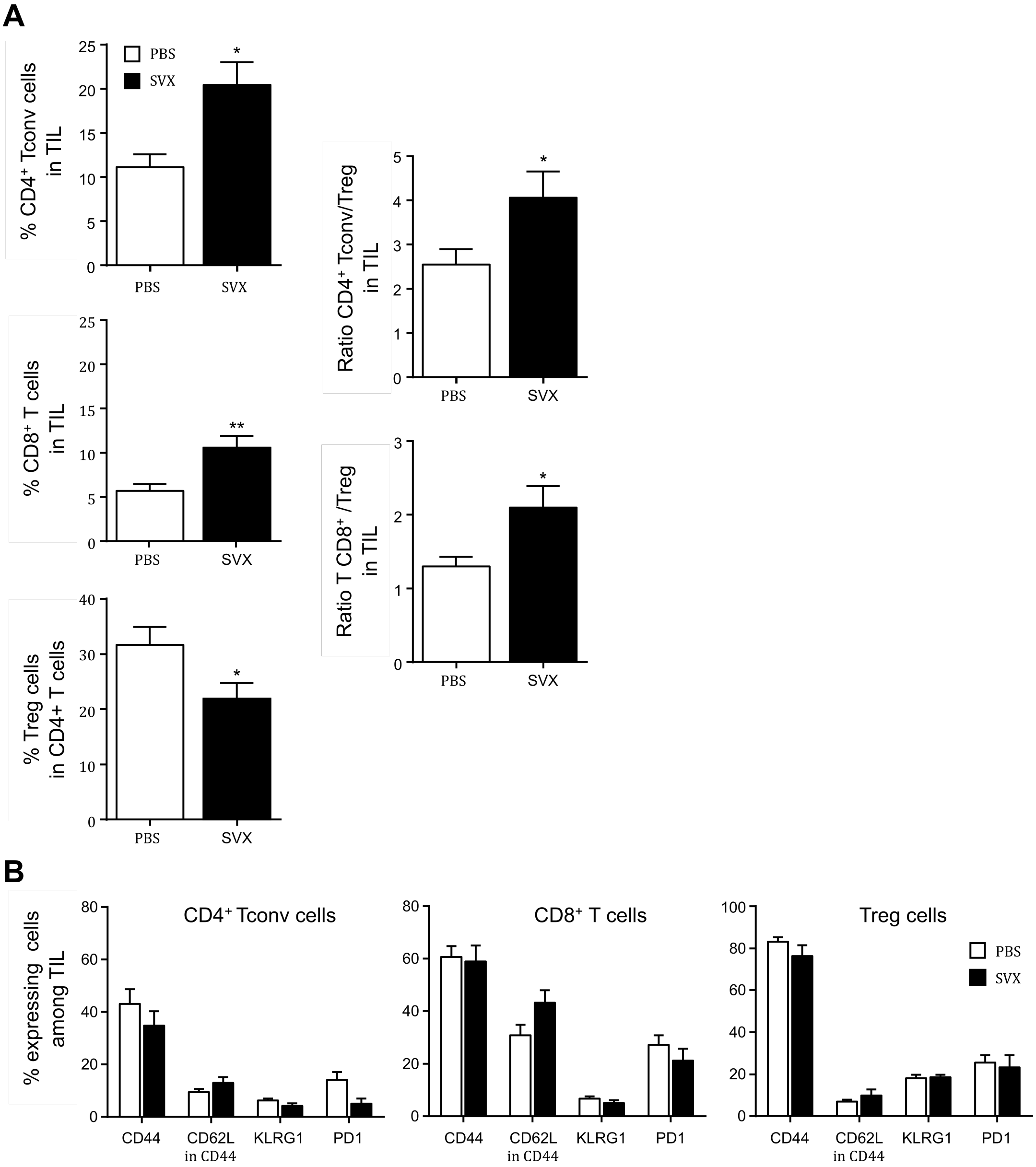

Supplementary Figure 3. SVX vaccine favors effector $CD4^+$ and $CD8^+$ T cells over Treg cells, in hA20 tumor-bearing mice. BALB/c mice were engrafted s.c with hA20 tumors and s.c injected with PBS or vaccinated with SVX (SVX). **(A)**. Two weeks after the last immunization, tumors were harvested, the immune cells were isolated, and the percentages of $CD4^+$ Tconv, $CD8^+$ T cells, and Treg cells, were evaluated in each individual mouse by flow cytometry. **(B)**. The expression of different cell surface markers was also evaluated in the different T-cell subsets isolated from the tumor. Data are presented as means of percentage of cells ± SEM of mice from two independent experiments (n=10 for SXV; n=16 for T). *P<0.05, **P<0.01 and *** P<0.001.
